# Otitis-Prone Children Produce Functional Antibodies to Pneumolysin and Pneumococcal Polysaccharides

**DOI:** 10.1128/CVI.00497-16

**Published:** 2017-03-06

**Authors:** Lea-Ann S. Kirkham, Selma P. Wiertsema, Karli J. Corscadden, Tulia Mateus, Gemma L. Mullaney, Guicheng Zhang, Peter C. Richmond, Ruth B. Thornton

**Affiliations:** a School of Paediatrics and Child Health, University of Western Australia, Perth, Australia; b Wesfarmers Centre for Vaccines and Infectious Diseases, Telethon Kids Institute, Perth, Australia; c School of Public Health, Curtin University, Perth, Australia; d Departments of Immunology and Paediatric Medicine, Princess Margaret Hospital for Children, Perth, Australia; Vanderbilt University Medical Center

**Keywords:** antibody potency, neutralizing titer, opsonophagocytosis, otitis media, pneumococcal conjugate vaccine, pneumococcal polysaccharides, pneumolysin, rAOM, Streptococcus pneumoniae, antibody function, vaccines

## Abstract

The pneumococcus is a major otitis media (OM) pathogen, but data are conflicting regarding whether otitis-prone children have impaired humoral immunity to pneumococcal antigens. We and others have shown that otitis-prone and healthy children have similar antibody titers to pneumococcal proteins and polysaccharides (vaccine and nonvaccine types); however, the quality of antibodies from otitis-prone children has not been investigated. Antibody function, rather than titer, is considered to be a better correlate of protection from pneumococcal disease. Therefore, we compared the capacities of antibodies from otitis-prone (cases) and healthy (controls) children to neutralize pneumolysin, the pneumococcal toxin currently in development as a vaccine antigen, and to opsonize pneumococcal vaccine and nonvaccine serotypes. A pneumolysin neutralization assay was conducted on cholesterol-depleted complement-inactivated sera from 165 cases and 61 controls. A multiplex opsonophagocytosis assay (MOPA) was conducted on sera from 20 cases and 20 controls. Neutralizing and opsonizing titers were calculated with antigen-specific IgG titers to determine antibody potency for pneumolysin, pneumococcal conjugate vaccine (PCV) polysaccharides, and non-PCV polysaccharides. There was no significant difference in antibody potencies between cases and controls for the antigens tested. Antipneumolysin neutralizing titers increased with the number of episodes of acute OM, but antibody potency did not. Pneumolysin antibody potency was lower in children colonized with pneumococci than in noncarriers, and this was significant for the otitis-prone group (*P* < 0.05). The production of functional antipneumococcal antibodies in otitis-prone children demonstrates that they respond to the current PCV and are likely to respond to pneumolysin-based vaccines as effectively as healthy children.

## INTRODUCTION

Otitis media (OM), middle ear infection, is responsible for the greatest numbers of general practitioner visits, antibiotic prescriptions, and surgical procedures for children in industrialized countries (1). Streptococcus pneumoniae (pneumococcus) is a major OM pathogen (1). Current pneumococcal conjugate vaccines (PCVs) are composed of capsule polysaccharides from up to 13 of the 95 immunologically distinct pneumococcal serotypes. PCVs have significantly reduced the prevalence of OM caused by the serotypes included in the vaccine (2, 3), but the overall reduction in the prevalence of OM has been negligible due to replacement disease with nonvaccine serotypes and other bacterial species (3–5). To address the limitations of serotype-specific vaccines, including the issue of replacement disease, research efforts are focusing on the development of pneumococcal vaccines that confer species-wide protection by using either whole-cell formulations or multicomponent recombinant pneumococcal proteins (6–11).

An attractive vaccine candidate is the highly conserved pneumococcal toxin pneumolysin (Ply). Immunization of animals with native or nontoxic derivatives of Ply elicits the production of neutralizing antibodies that confer serotype-independent protection from pneumococcal pneumonia and bacteremia (12–15). Recent clinical trials with Ply-based vaccines have demonstrated that they are safe (16, 17) and elicit high circulating titers of neutralizing anti-Ply antibodies in humans (16). Ply-induced protection against OM in humans remains to be demonstrated for these vaccines, but the fusion of choline binding protein A (CbpA) peptides to a Ply toxoid has been shown to enhance protection against pneumococcal OM in mice (11). The role of Ply in pneumococcal OM is not fully understood, but direct instillation of Ply into the cochlea of guinea pigs damages the inner and outer hair cells (18), suggesting that Ply may contribute to permanent hearing loss, which can occur in severe cases of pneumococcal OM. Ply is involved in early biofilm development (19), a key feature of OM pathogenesis (20) that contributes to the recurrence of infections and bacterial resistance to antibiotic treatment. Together, these data indicate that Ply-containing vaccines may have the potential to reduce the burden of pneumococcal OM.

Pneumococcal carriage and acute OM (AOM) induce local and systemic production of anti-Ply and anticapsule antibodies in children within the first years of life (21–28). It has been suggested that children with recurrent episodes of OM (otitis prone) have impaired naturally acquired and vaccine-induced antibody responses to pneumococcal antigens, with reports of lower anti-Ply IgG (21), anticapsule IgG (23), IgG2, and IgA (23) titers. In contrast, we and others observed that titers of anti-Ply IgG (25, 28) and anticapsule polysaccharide IgG, IgG2, and IgA (29–32) in sera from otitis-prone children were similar to or even higher than those in sera from non-otitis-prone children. Previous studies of humoral immunity in otitis-prone children assessed antibody titer rather than function, but high titers of antipneumococcal polysaccharide antibodies do not necessarily correlate with antibody function (33, 34) or protection from disease (35). We hypothesized that although otitis-prone children may produce similar serum IgG titers to pneumococcal vaccine and nonvaccine antigens, the functionality of these antibodies may be reduced and thus responsible for susceptibility to recurrent OM. We used a Ply neutralization assay (36) and the multiplexed quadruple-serotype opsonophagocytic killing assay (MOPA4) (37) to compare the functional properties of anti-Ply and anticapsule antibodies in otitis-prone and healthy children. Investigation into antibody quality in otitis-prone children is important for evaluating current vaccine strategies and the suitability of a Ply-based vaccine to reduce the burden of pneumococcal OM.

## RESULTS

### Study population.

Table 1 details host and environmental risk factors for children in this study. Age, gender, and day care attendance were adjusted for in all analyses, as we previously found that they are confounding factors for pneumococcal polysaccharide and protein IgG titers in this cohort (28, 29). Ply IgG antibody titers and neutralizing titers (NTs) were measured in serum samples from 165 cases and 61 controls. The mean age of cases was higher than that of healthy controls (21.2 months versus 18.7 months; *P* = 0.03). Although males are more likely to be otitis prone than females, there was no significant difference in gender distribution in this cohort (62% of cases and 72% of controls were male; *P* = 0.15). Otitis-prone children were significantly more likely to attend a day care facility for ≥4 h/week than were healthy controls (62% versus 32%; *P* < 0.001). Almost all children were fully vaccinated with the 7-valent PCV (PCV7) (99% of cases and 97% of controls). Rates of antibiotic use were similar, with 22% of cases and 20% of controls taking antibiotics at the time of surgery (*P* = 0.77). Otitis-prone children in the Ply study had experienced an average of 7.3 AOM episodes, with 33% of children having had 3 or 4 episodes, 18% having had 5 to 7 episodes, 35% having had 8 or 9 episodes, and 15% having experienced more than 10 AOM episodes. Significantly more cases were colonized with S. pneumoniae at the time of sample collection than controls (42% versus 26%; *P* = 0.03).

**TABLE 1:** Host and environmental risk factors for otitis-prone and healthy children assessed by a Ply neutralization assay and a MOPA

Parameter	Ply neutralization assay			MOPA		
	Value for group		*P* value	Value for group		*P* value
	Otitis prone (*n* = 165)	Healthy (*n* = 61)		Otitis prone (*n* = 20)	Healthy (*n* = 20)	
Mean age (mo) (range)	21.2 (7.3–36.0)	18.7 (7.3–35.0)	0.03	21.0 (10.9–33.5)	18.3 (7.0–35.0)	0.07
% male subjects	62	72	0.15	65	85	0.14
% of subjects at day care ≥4 h/wk	62^*a*^	32^*b*^	<0.001	50	30	0.20
% of subjects fully PCV7 vaccinated	99	97	0.30	100	100	
% of subjects taking antibiotics	22^*c*^	20^*b*^	0.77	0	0	
% of subjects with no. of AOM episodes						
3–4	33			55		
5–7	18			10		
8–9	35			25		
10+	15			10		
% of subjects with pneumococcal carriage	42	26	0.03	50	35	0.34

a *n* = 151.

b *n* = 59.

c *n* = 152.

A MOPA was conducted on a subset of sera from 20 cases and 20 controls. No differences in risk factors between the cases and controls were observed (Table 1). Antibiotic use and PCV7 vaccination affect the opsonophagocytosis assay; therefore, children were excluded if they had received antibiotics within the month prior to surgery, and all children were fully PCV7 vaccinated. Otitis-prone children in the MOPA study experienced an average of 5.6 AOM episodes, with 55% of children having had 3 or 4 episodes, 10% having had 5 to 7 episodes, 25% having had 8 or 9 episodes, and 10% having experienced more than 10 AOM episodes. There was no significant difference in the numbers of cases and controls who were colonized with S. pneumoniae at the time of sample collection (50% versus 35%; *P* = 0.34).

### Potencies of anti-Ply neutralizing antibodies of otitis-prone and healthy children are similar.

**FIG 1:**
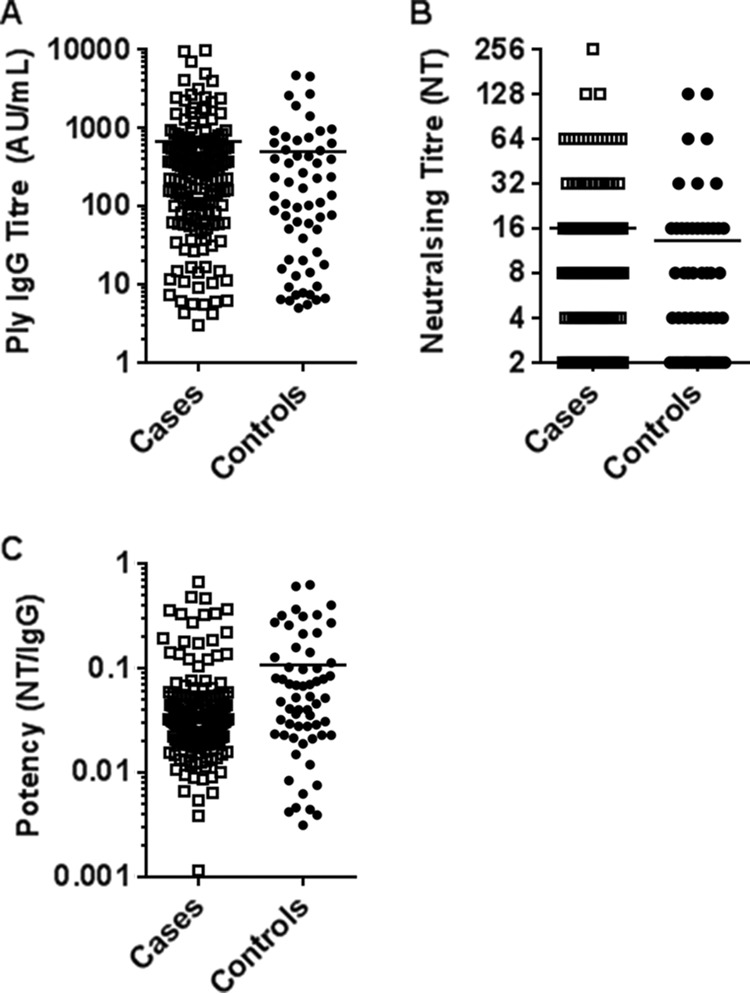
Pneumolysin IgG antibody titers (A), neutralizing titers (NTs) (B), and antibody potencies (C) in sera from otitis-prone (open squares) and healthy (closed circles) children. Data are presented for each individual child, with the horizontal bars depicting the means. Statistical analysis was conducted on the geometric mean of log-transformed data, correcting for age, gender, and day care attendance. AU, arbitrary units; Ig, immunoglobulin.

**FIG 2:**
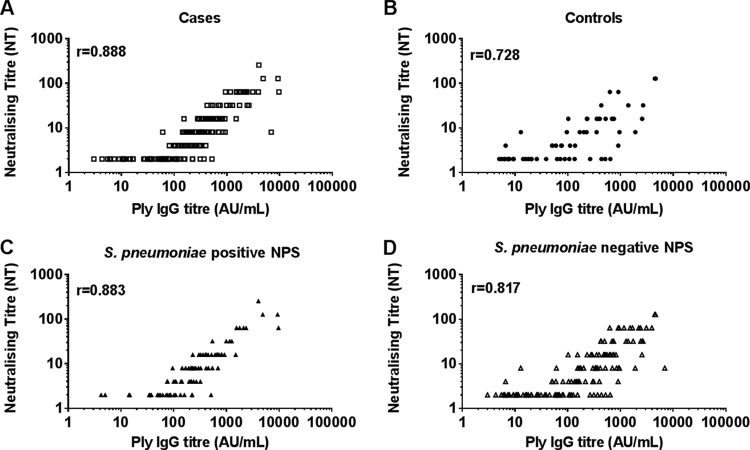
Correlation between pneumolysin (Ply) IgG titers (AU per milliliter) and neutralizing titers (NTs) for sera from otitis-prone (A) and healthy (B) children and children colonized (C) and not colonized (D) with S. pneumoniae. In all groups, there was a strong and significant correlation between Ply IgG titers and NTs (*P* < 0.0001). NPS, nasopharyngeal swab.

The geometric mean Ply IgG titers (Fig. 1A), neutralizing titers (Fig. 1B), and antibody potencies (Fig. 1C) were similar between otitis-prone and healthy children (*P* = 0.394, *P* = 0.592, and *P* = 0.069, respectively). There was a strong and significant correlation between Ply IgG titers and neutralizing titers in all cohorts (*r* = 0.842; *P* < 0.001). This significant positive correlation remained when cases and controls were assessed separately (*r* = 0.888 and *r* = 0.728, respectively; *P* < 0.001) and also for those colonized or not colonized with S. pneumoniae (*r* = 0.883 and *r* = 0.817, respectively; *P* < 0.001) (Fig. 2). Colonizing serotypes were identified for all children in this study who had S. pneumoniae isolated from their nasopharyngeal swabs. The isolates were mostly nonvaccine serotypes, with the most common serotypes isolated from cases being serotypes 19A (*n* = 23), 11A (*n* = 9), 6A (*n* = 5), and 15B (*n* = 5), while the most common colonizing serotypes in controls were serotypes 19A (*n* = 5), 6A (*n* = 4), and 23B (*n* = 2). The colonizing serotype did not influence the potency of Ply antibodies, but this would be expected, as Ply is conserved and expressed by virtually all serotypes.

### The number of AOM episodes experienced does not affect anti-Ply antibody potency.

**FIG 3:**
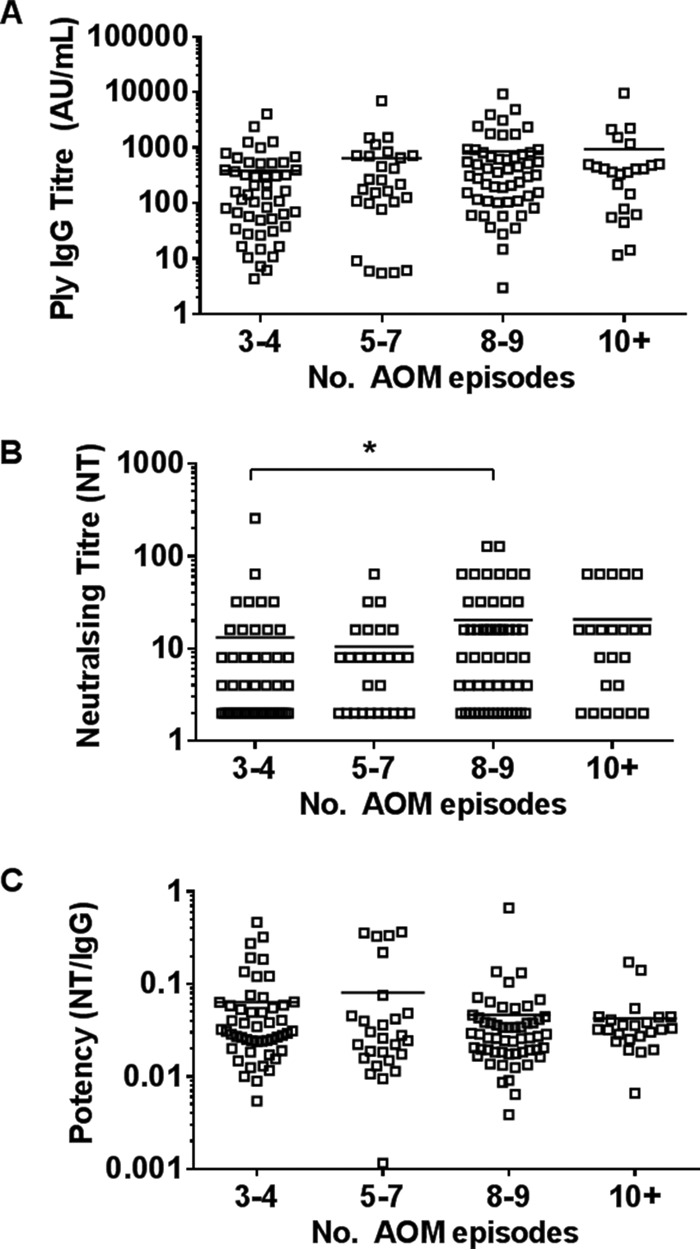
Pneumolysin IgG antibody titers (A), neutralizing titers (B), and antibody potencies (C) in sera from otitis-prone children according to the number of AOM episodes experienced. Data are presented for each individual child, with the horizontal bars depicting the means. Statistical analysis was conducted on the geometric mean of log-transformed data, correcting for age, gender, and day care attendance. *, *P* < 0.05.

Increasing numbers of AOM episodes resulted in an overall trend toward higher anti-Ply IgG titers (Fig. 3A) and neutralizing titers (Fig. 3B), but overall, there was no difference in anti-Ply antibody potency (Fig. 3C) regardless of the number of AOM episodes. When raw data were corrected for confounding factors, including age, which differed between cases in the different categories of numbers of AOM episodes, children experiencing 10 or more AOM episodes had a downward but not significant trend in the geometric mean anti-Ply IgG titer (207 arbitrary units [AU]/ml) compared with children experiencing 8 or 9 AOM episodes (260 AU/ml) and had anti-Ply IgG titers that were similar to those for children experiencing 5 to 7 AOM episodes (209 AU/ml). There was a significant increase in the adjusted geometric mean neutralizing titer of sera from children with 8 or 9 episodes of AOM compared to those for children with 3 or 4 episodes (*P* < 0.05), but this significance did not remain when antibody potency was calculated.

### Otitis-prone children colonized with S. pneumoniae have high circulating levels of poor-quality anti-Ply antibody.

**FIG 4:**
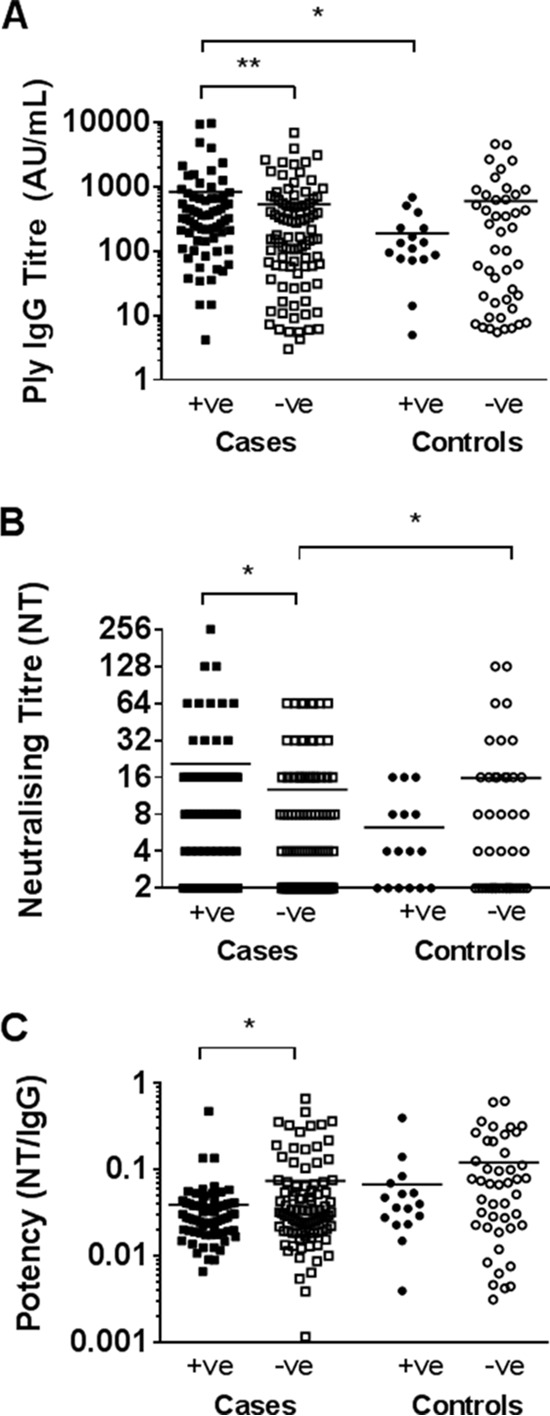
Pneumolysin antibody IgG titers (A), neutralizing titers (B), and antibody potency (C) in sera from otitis-prone (cases) and healthy (controls) children according to nasopharyngeal colonization with S. pneumoniae. Data are presented for each individual child, with the horizontal bars depicting the means. Statistical analysis was conducted on the geometric mean of log-transformed data, correcting for age, gender, and day care attendance. Closed data points represent children colonized with S. pneumoniae, and open data points represent noncolonized children. **, *P* < 0.001; *, *P* < 0.05.

Otitis-prone children colonized with S. pneumoniae at the time of sample collection (*n* = 70) had significantly higher anti-Ply IgG (Fig. 4A) and Ply-neutralizing (Fig. 4B) titers but lower antibody potency (Fig. 4C) than did noncolonized otitis-prone children (*n* = 95) (*P* < 0.01, *P* = 0.01, and *P* = 0.046, respectively). This difference was not observed in healthy children, where anti-Ply antibody titers, neutralizing titers, and potencies were similar between colonized (*n* = 16) and noncolonized (*n* = 45) controls (Fig. 4). The anti-Ply IgG titer was significantly higher in S. pneumoniae-colonized cases than in colonized controls (*P* = 0.01) (Fig. 4A), whereas the neutralizing titer was significantly higher in noncolonized controls than in noncolonized cases (*P* = 0.01) (Fig. 4B). There was no significant difference in antibody potency between colonized cases and controls or between noncolonized cases and controls (Fig. 4C).

### Potencies of antibodies against pneumococcal capsule polysaccharides are similar between otitis-prone and healthy children.

**FIG 5:**
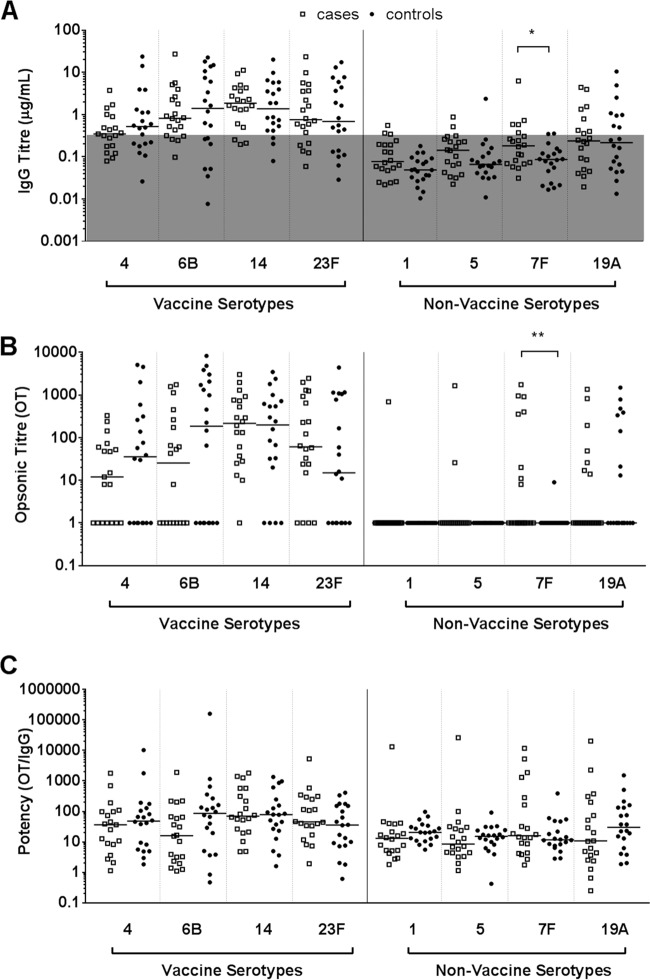
Pneumococcal polysaccharide (Pn) IgG antibody titers (A), opsonization titers (OTs) (B), and antibody potency (C) in sera from otitis-prone (open squares) and healthy (closed circles) children. Data are presented for each individual child, with the horizontal bars depicting the means. Statistical analysis was conducted on the geometric mean of log-transformed data, correcting for age, gender, and day care attendance. *, *P* < 0.05 for comparison of cases to controls for each polysaccharide. The gray shading in panel A indicates the IgG level (0.35 μg/ml) above which polysaccharide IgG titers are considered to provide protection against pneumococcal disease.

There was no significant difference between cases and controls in anti-capsular polysaccharide IgG concentrations (Fig. 5A) and opsonization titers (OTs) (Fig. 5B) for 7 of the 8 pneumococcal polysaccharides tested. The serotype 7F IgG concentration and opsonization titer were significantly higher in the cases than in the controls (*P* = 0.03 and *P* = 0.02, respectively). However, when antibody potency was calculated (opsonization titer/IgG titer), there was no significant difference between cases and controls for any of the pneumococcal polysaccharide antibodies (Fig. 5C).

Both cases and controls had higher antibody titers, opsonization titers, and antibody potencies against vaccine serotypes than against the nonvaccine serotypes (Fig. 5). The geometric mean IgG concentration and opsonizing titer for antibodies against all PCV7 serotypes tested were above the conservative correlates of protection of 0.35 μg/ml IgG (38) (Fig. 5A) and an opsonization titer of >8 (34) (Fig. 5B), respectively, for all children, regardless of OM status.

## DISCUSSION

This is the first investigation into whether otitis-prone children have deficiencies in their functional antibody responses to pneumococcal antigens. We have shown that otitis-prone children produce neutralizing antibodies to the pneumococcal toxin Ply and opsonizing antibodies to vaccine and nonvaccine capsule polysaccharides. The potency of these antibodies was comparable to that of antibodies produced by healthy age-matched controls, indicating that antipneumococcal antibody function is not impaired in otitis-prone children. Whether this is true for the stringently defined otitis-prone child, in which anti-Ply IgG titers are lower than those in non-otitis-prone children (22), remains to be determined.

The neutralizing-antibody assay is the standard test to assess functional antibody responses to vaccines containing Ply. As current phase II clinical trials with a Ply-containing pneumococcal conjugate vaccine (17) and a whole-cell pneumococcal vaccine are under way (ClinicalTrials.gov registration no. NCT02097472), the anti-Ply neutralization test will become even more relevant for determining the immunogenicity of Ply. While numerous studies have assessed neutralizing activity in serum following immunization of animals with Ply (12, 14, 36), this is the first report describing Ply-neutralizing titers in children and only the second report describing them in humans. The first assessment of human anti-Ply neutralizing antibodies was from a recent phase I clinical trial with a Ply toxoid (PlyD1) in adults (16). That trial demonstrated that injection of PlyD1 was tolerated and induced neutralizing antibody titers that were 4-fold higher than baseline titers, thereby validating the use of Ply as a vaccine antigen. Interestingly, the authors of that study found only a weak to moderate association between Ply IgG titers and neutralizing titers in placebo adult sera (*r* = 0.490) compared to the strong correlation that we observed for the children in our study (*r* = 0.842; *P* < 0.001). This may suggest that naturally acquired anti-Ply antibodies are more functional and of higher quality in children than in adults. Indeed, opsonizing activity, but not the concentration of anticapsule antibodies, has been shown to diminish with age (33). It is important to note that Vero cell cytotoxicity rather than inhibition of hemolysis was used in the PlyD1 study; therefore, neutralizing titers cannot be directly compared between studies.

Consideration of the timing of sample collection and the number of previous AOM episodes is important for comparison of anti-Ply antibody titers and function between otitis-prone and healthy children, as anti-Ply serum IgG titers may diminish during an episode of pneumococcal AOM in naive children but not in children with multiple AOM episodes (21, 25). Another possibility is that recurrent AOM may induce antigen-specific immune tolerance, as indicated in this study, where children experiencing 10 or more AOM episodes had lower age-adjusted geometric mean anti-Ply IgG titers than did children experiencing 8 or 9 AOM episodes. Our data indicate that it is also important to know whether children are colonized with S. pneumoniae at the time of serum collection, as anti-Ply antibody titers were higher in otitis-prone children that were colonized with S. pneumoniae, but the potency of these antibodies was lower than that of antibodies in noncolonized otitis-prone children. This may be due to the generation of low-affinity antibodies as part of a primary mucosal response to the colonization event and prior to avidity maturation. Whether anti-Ply antibodies are protective against pneumococcal OM remains to be determined. It is also possible that pneumococcal colonization (and disease) induces the production of ineffective anti-Ply antibodies, possibly to decoy epitopes, particularly in children experiencing recurrent episodes of pneumococcal AOM. Future studies involving epitope mapping of anti-Ply antibodies from otitis-prone and healthy children are warranted, particularly in the context of using Ply-based vaccines for otitis-prone children.

To validate our finding that anti-Ply antibody function was not impaired in otitis-prone children, we also measured the opsonizing activity of antibodies to vaccine and nonvaccine pneumococcal polysaccharides in a subset of children by using a MOPA (34). The MOPA is considered to be the gold standard to measure anticapsule antibody quality and is recommended by the WHO for full evaluations of pneumococcal conjugate vaccine efficacy (39). The demonstration that otitis-prone children in our study produced anti-capsule polysaccharide antibody that was of a quality similar to that of healthy controls, both in response to PCV7 vaccination and from natural acquisition, adds to the increasing evidence that humoral immunity may not be impaired in otitis-prone children. Indeed, evidence is mounting that cell-mediated immune defenses and mucosal antibody, rather than circulating antibody, may be important for limiting pneumococcal colonization, transmission, and OM (40–44). The higher opsonizing titers observed for serotype 7F IgG in cases than in controls are not able to be explained in this study. Serotype 7F, and the cross-protective serotype 7A, was not isolated from this cohort, indicating that this serotype was not circulating at the time of sample collection (45).

In summary, we have shown that the potencies of antibodies to pneumococcal vaccine and nonvaccine antigens are similar in otitis-prone and healthy children. This suggests that otitis-prone children respond as well as their healthy counterparts to current PCV immunizations and should also respond well to immunization with Ply-derived vaccines. Whether protein-based pneumococcal vaccines protect against OM remains to be determined.

## MATERIALS AND METHODS

### Recruitment of the study cohort and sample collection.

Recruitment of children to this cross-sectional study (the GROMIT study) was described previously (45). Briefly, recruitment was conducted from 2007 to 2009, and children were aged 6 to 36 months. Cases were recruited at the time of surgery and were defined as children with a history of at least 3 episodes of AOM in 6 months or 4 episodes in 12 months who required the insertion of ventilation tubes. Children with no significant history of OM and who were undergoing general surgery for noninfectious reasons were recruited as healthy age-matched controls. None of the children had signs of acute infection at the time of sample collection. Children with diagnosed immunodeficiency, cystic fibrosis, immotile-cilia syndrome, craniofacial abnormalities, and chromosomal or genetic syndromes were excluded. Data on ear disease and host and environmental risk factors were collected by using parental questionnaires and from medical records. Nasopharyngeal swabs and serum samples were collected from all cases and controls as previously described (45). This study was approved by the Princess Margaret Hospital for Children Human Research Ethics Committee, Perth, Western Australia, and by the institutional boards of the private hospitals where recruitment took place.

### Measurement of anti-Ply IgG and pneumococcal polysaccharide IgG titers.

Serum anti-Ply IgG antibody titers were measured with a bead-based immunoassay as previously described (28). Serum antipolysaccharide IgG titers to PCV7 vaccine serotypes 4, 6B, 14, and 23F and non-PCV7 vaccine serotypes 1, 5, 7F, and 19A were also measured by using a multiplex bead-based immunoassay as previously described (29).

### Cholesterol depletion and complement inactivation of serum.

Free cholesterol, which blocks the cytolytic activity of Ply, was depleted from sera by the addition of 10% (vol/vol) cholesterol depletion working solution (10 g/liter dextran sulfate–0.5 M MgCl_2_ in distilled water) (46) (all from Sigma-Aldrich). Samples were then vortexed, incubated for 15 min at room temperature (RT), and then centrifuged at 1,500 × *g* for 30 min at RT. Cholesterol-free serum (supernatant) was transferred to a fresh tube and incubated at 56°C for 30 min to inactivate serum complement. Cholesterol depletion was confirmed for a subset of serum samples (*n* = 10) by using the Vitros Chol slide system (Ortho Clinical Diagnostics, Rochester, NY, USA).

### Ply neutralization assay.

The ability of cholesterol-depleted complement-inactivated sera to neutralize the cytolytic activity of Ply was measured as previously described (12). Briefly, cholesterol-depleted complement-inactivated serum samples were serially diluted 1:2 in phosphate-buffered saline (PBS; Sigma) in a U-bottom 96-well plate. The hemolytic activity of recombinant Ply was measured (14), and 50 μl of 80 hemolytic units (HU)/ml of Ply was added to each well to give 4 HU/well. The plate was incubated at 37°C for 15 min to allow anti-Ply antibodies to bind to and potentially neutralize Ply. A 1% washed human red blood cell solution in PBS was added to reach a final concentration of 0.25% in each well. The plate was incubated at 37°C for 30 min and centrifuged for 5 min at 200 × *g* at RT to facilitate the precipitation of intact erythrocytes. The endpoint of the assay (neutralizing titer [NT]) was taken as the reciprocal of the serum dilution at which the complete inactivation of 4 HU of Ply was observed (no hemolysis). An adult reference serum sample, a negative hemolysis control (saline), and a positive hemolysis control (4 HU Ply) were run on each plate.

To confirm that the observed neutralizing activity was antibody mediated, IgG was depleted from the adult reference serum sample by using protein G-Sepharose columns (Invitrogen, Victoria, Australia), and serum neutralizing titers before and after IgG depletion were compared. IgG depletion in serum was confirmed by nephelometry (data not shown).

The potency of the anti-Ply antibodies present was calculated as the ratio of the neutralizing titer to the anti-Ply IgG antibody concentration and gives a measure of the quality of anti-Ply antibodies in serum. Potency has previously been used to describe the quality of antipolysaccharide responses in PCV-vaccinated adults (47).

### Multiplexed quadruple-serotype opsonophagocytic killing assay.

A MOPA was conducted as previously described (37), using eight antibiotic-resistant pneumococcal serotypes (serotypes 1, 4, 5, 6B, 7F, 14, 19A, and 23F), which were kindly provided by Moon Nahm. Each serotype was resistant to one of four antibiotics (optochin, streptomycin, spectinomycin, and trimethoprim) and susceptible to the other three. A detailed protocol is available at the Bacterial Respiratory Pathogen Laboratory website (see http://www.vaccine.uab.edu/). A MOPA was conducted on a subset of sera from 20 children with a history of recurrent AOM (cases) and 20 healthy controls due to limitations in the available sample volumes and the impact of antibiotic use on MOPA results. To assess potential differences in natural versus vaccine-elicited responses in otitis-prone children compared to healthy controls, a MOPA was conducted on 4 selected PCV7 serotypes and 4 non-PCV7 serotypes. Opsonization titers (OTs) were expressed as the serum dilution that kills 50% of cells of the specific pneumococcal serotype. Serum samples were used at a 1:2 dilution in opsonization buffer B and then underwent 3-fold dilutions to reach a final dilution of 1:26,000. Samples with a titer below the detection limit of 2 were assigned a value of 1 for data analysis.

The potency of the antipolysaccharide antibodies was calculated as the ratio of the opsonization titer to the antipolysaccharide IgG antibody titer for each pneumococcal serotype tested (47). This gives a measure of the quality of opsonizing antibodies in each serum sample.

### Statistical analyses.

Host and environmental risk factors between cases and controls were compared by using Student's *t* tests for continuous variables (age) and Pearson chi-square analyses (*P* value, asymptotic significant, 2-sided) for categorical variables (gender, day care attendance, PCV7 vaccination status, current antibiotic use, AOM category, and pneumococcal carriage). Spearman's rank correlation coefficient was used to determine the correlation between anti-Ply IgG titers and neutralizing titers. A general linear regression model (on log-transformed data and adjusting for confounders of age, gender, and day care attendance) was used to analyze differences in antipneumococcal (anti-Ply and serotype-specific antipolysaccharide IgG) antibody levels between otitis-prone and non-otitis-prone children. The IBM SPSS Statistics 22 for Windows software package (IBM, New York, NY, USA) was used for all statistical analyses, and data were plotted by using GraphPad Prism 6 (GraphPad Software Inc., CA, USA).
